# Predicting Oral Food Challenge Outcomes in Children with Suspected Cow’s Milk Allergy: Clinical Utility of Casein-Specific IgE and Skin Test Ratios

**DOI:** 10.3390/nu18132187

**Published:** 2026-07-05

**Authors:** Filiz Demir Şahin, Hilal Şahin Sindi, Ozan Kapçay, Mehmet Kılıç

**Affiliations:** Department of Pediatric Allergy and Immunology, Faculty of Medicine, Fırat University, Elazığ 23119, Türkiye; hilalsahin.dr@gmail.com (H.Ş.S.); ozankapcay@gmail.com (O.K.); drmkilic@gmail.com (M.K.)

**Keywords:** Cow’s milk allergy, oral food challenge, casein-specific IgE, skin prick test, children

## Abstract

Background: To investigate the diagnostic value of the SPT-to-histamine ratio, casein-specific immunoglobulin E (IgE), and cow’s milk-specific IgE in predicting oral food challenge (OFC) positivity in children with suspected cow’s milk allergy. Methods: In this single-center retrospective observational study, 126 children evaluated for suspected cow’s milk allergy who underwent OFC between January 2019 and July 2024 at a tertiary pediatric allergy and immunology center were included. Demographic and clinical characteristics, SPT parameters, and laboratory biomarkers were analyzed. Discriminatory performance was assessed using receiver operating characteristic (ROC) analysis, and independent predictors were identified using multivariable logistic regression. Results: OFC was positive in 66 patients (52.4%). Casein-specific IgE demonstrated strong discriminatory performance (AUC = 0.878), with 80.3% sensitivity and 85.0% specificity at a cutoff value of ≥2.55 kUA/L. The SPT-to-histamine ratio showed moderate discriminatory performance (AUC = 0.758). In multivariable analysis, only casein-specific IgE remained an independent predictor of OFC positivity (adjusted OR = 1.317, *p* < 0.001). Conclusions: Casein-specific IgE is a strong independent predictor of OFC positivity. Although SPT-derived ratios may provide complementary diagnostic information, their independent predictive contribution appears limited. These findings support the role of component-resolved diagnostics in pre-challenge risk stratification.

## 1. Predicting Oral Food Challenge Outcomes in Children with Suspected Cow’s Milk Allergy: Clinical Utility of Casein-Specific IgE and Skin Test Ratios

Cow’s milk allergy (CMA) is one of the most common food allergies in early childhood and remains a significant clinical problem, particularly in infants and young children. Its clinical manifestations may involve the skin, gastrointestinal tract, respiratory system, and less frequently the cardiovascular system, resulting in a broad and heterogeneous spectrum of presentations. This variability often complicates the diagnostic process. The prevalence of CMA in children has been estimated to range between approximately 0.5% and 3%, particularly during the first year of life, underscoring its clinical relevance in pediatric allergy practice [1].

The diagnostic evaluation of CMA primarily relies on an allergy-focused clinical history, skin prick testing (SPT), and measurement of serum-specific IgE. However, while these tests reflect sensitization, they do not always correlate with true clinical reactivity [2]. Therefore, especially in cases with inconclusive clinical history or discordant test results, the oral food challenge (OFC) remains the reference standard for confirming the diagnosis [3]. Current European Academy of Allergy and Clinical Immunology guidelines emphasize that the evaluation of suspected food allergy should begin with a detailed clinical history supported by SPT and/or specific IgE testing, with OFC playing a central role when confirmation of clinical reactivity is required [4,5].

In recent years, component-resolved diagnostics, particularly casein-specific IgE, have gained increasing attention in the assessment of CMA. Several studies have suggested that sensitization to individual milk proteins, especially casein, may be associated not only with OFC outcomes but also with a more persistent disease course. However, the diagnostic performance of casein-specific IgE has shown considerable variability across studies, likely reflecting differences in age, clinical phenotype, study design, and assay methodologies [6,7]. Consequently, although casein-specific IgE appears to be a promising biomarker, its role in predicting OFC positivity in routine clinical practice remains incompletely defined.

In parallel, alternative SPT-derived parameters have been proposed to improve the interpretation of cutaneous responses [8]. Among these, ratios normalized to the histamine wheal response may provide a more individualized assessment by partially accounting for inter-individual variability in skin reactivity. Nevertheless, the incremental value of such indices over conventional SPT wheal size and serum-based biomarkers has not been fully established. In particular, data simultaneously evaluating SPT-to-histamine ratios alongside casein-specific IgE and other laboratory biomarkers within the same pediatric cohort remain limited.

Moreover, unnecessary elimination diets in children with suspected CMA may increase the risk of nutritional deficiencies, impair normal growth, and impose an unnecessary burden on affected children and their families, underscoring the importance of establishing an accurate diagnosis before recommending long-term dietary restrictions [2]. Therefore, improving the prediction of OFC outcomes may not only reduce the number of unnecessary challenge procedures but also contribute to more appropriate dietary management and avoidance of prolonged dietary restrictions. In this context, clinically useful biomarkers that support risk stratification before OFC may have important implications for nutritional management in pediatric patients.

Given these considerations, further studies integrating multiple skin test– and serum-based biomarkers within the same patient population are warranted to improve the prediction of OFC outcomes and to support more accurate risk stratification in clinical practice. Accordingly, the present study aimed to evaluate the association between selected clinical and laboratory parameters and OFC positivity in children with suspected CMA. Specifically, we investigated the predictive value of age, cow’s milk SPT wheal diameter, histamine response, SPT-to-histamine ratio, casein-specific IgE, cow’s milk-specific IgE, and total IgE for OFC positivity.

## 2. Materials and Methods

### 2.1. Study Design and Population

This single-center, retrospective observational study was conducted at the Department of Pediatric Allergy and Immunology, Fırat University Hospital, Elazığ, Türkiye, and included pediatric patients evaluated for suspected cow’s milk allergy between January 2019 and July 2024.

Medical records were systematically reviewed, and all consecutive patients who met the inclusion criteria during the study period were considered eligible to minimize selection bias. A total of 126 children with complete clinical data who underwent standardized skin prick testing (SPT) with cow’s milk extract and oral food challenge (OFC) based on clinical indications were included in the study.

Patients with a well-documented history of cow’s milk-induced anaphylaxis, confirmed primary or secondary immunodeficiency, or the presence of an acute infection at the time of OFC were excluded.

### 2.2. Skin Prick Testing

Skin prick testing was performed on the volar surface of the forearm using a standardized commercial cow’s milk allergen extract (Asacpharma, ApiPrick^®^, Alicante, Spain) and single-use applicators (Medblue Allergy, Asistan Medikal, Gaziantep, Türkiye). Histamine dihydrochloride (10 mg/mL) and 0.9% sodium chloride solution served as the positive and negative controls, respectively. Wheal diameters were measured 15 min after application in millimeters, and the largest diameter was recorded. A positive SPT response was defined as a wheal diameter at least 3 mm greater than that of the negative control, in accordance with current international recommendations [9]. SPT reactivity was further evaluated using the cow’s milk wheal diameter, histamine wheal diameter, and the SPT-to-histamine ratio. Antihistamine medications were withheld for an appropriate period prior to testing in accordance with current clinical practice recommendations. All procedures were performed under standardized conditions by experienced pediatric allergy and immunology staff.

### 2.3. Oral Food Challenge (OFC)

The OFC was performed to assess clinical reactivity to cow’s milk, based on the recommendations of the European Academy of Allergy and Clinical Immunology (EAACI) and the PRACTALL consensus, as adapted to our center’s clinical practice protocol. All challenges were conducted in a hospital setting under physician supervision. OFC was performed within 1 month after skin prick testing and serum-specific IgE measurement.

Before testing, all patients were evaluated after an age-appropriate fasting period, typically 4–6 h. Patients were systematically assessed for active infection, uncontrolled or moderate-to-severe persistent asthma, a history of anaphylaxis within the preceding 4–6 weeks, significant cardiovascular disease, and the use of beta-blockers or angiotensin-converting enzyme inhibitors. Patients meeting any of these criteria did not undergo OFC and were excluded from the study. In addition, antihistamines and other medications that could affect test results were discontinued before testing according to recommended washout periods.

The challenge material was standardized according to age: commercial cow’s milk-based infant formula was used in children younger than 1 year, whereas pasteurized cow’s milk was used in children aged 1 year or older. OFC was performed using a 6-step dose-escalation protocol adapted to our center, based on the semi-logarithmic incremental dosing model recommended in the EAACI/PRACTALL guidelines [4] (Table 1). Observation intervals of 20–30 min were maintained between doses. The starting dose and dose-escalation schedule were individualized based on each patient’s clinical history and risk profile. In patients considered at higher risk for anaphylaxis, lower starting doses were used, dose escalation was performed more gradually, and intermediate steps were added when necessary.

The cumulative target dose was determined according to age: 100–150 mL for children younger than 1 year and 200–220 mL for those aged 1 year or older (Table 1). These doses were based on our institutional OFC protocol and were intended to approximate an age-appropriate serving of cow’s milk.

During the challenge, patients were closely monitored for objective allergic manifestations after each administered dose. The test was terminated and considered positive upon the development of objective clinical findings. Positivity criteria included cutaneous manifestations (urticaria, angioedema), gastrointestinal symptoms (vomiting, abdominal pain, diarrhea), respiratory symptoms (cough, wheezing, dyspnea), and cardiovascular findings (hypotension, syncope). Isolated subjective symptoms were not considered sufficient for a positive test result.

Patients who completed the planned cumulative dose without developing symptoms and who demonstrated tolerance to cow’s milk during at least 2 h of post-challenge observation and subsequent feeding were classified as OFC-negative.

All challenges were performed in a fully equipped clinical setting with immediate availability of emergency medications and resuscitation equipment, including intramuscular epinephrine. Following completion of the test, all patients were observed for at least 2 h to monitor for delayed reactions. All oral food challenges were performed under the direct supervision of experienced pediatric allergy specialists. Throughout the procedure, patients were continuously monitored for both subjective and objective symptoms. Mild transient subjective symptoms without accompanying objective findings (e.g., isolated perioral itching) were observed and did not automatically result in challenge termination. The challenge was discontinued only when predefined objective allergic reactions occurred or when symptoms progressed according to institutional stopping criteria.

### 2.4. Data Collection

Medical records were systematically reviewed, and data were extracted using a standardized data collection form. The following variables were recorded: demographic characteristics (age and sex); presenting symptoms; clinical reaction phenotypes; comorbid atopic diseases (atopic dermatitis, asthma, and allergic rhinitis); history of cow’s milk–related adverse reactions; skin test parameters (SPT wheal diameter, histamine wheal diameter, and SPT-to-histamine ratio); laboratory parameters (cow’s milk-specific IgE, casein-specific IgE, total IgE, and the specific IgE/total IgE ratio); and OFC outcomes.

### 2.5. Outcome Measures

#### Primary Outcome

The primary outcome was defined as early-type clinical reactivity during the OFC or the immediate observation period, characterized by the development of objective clinical findings. Objective positivity criteria included urticaria, angioedema, vomiting, bronchospasm, anaphylaxis, or generalized pruritus accompanied by objective signs. The occurrence of any of these findings was considered a positive OFC result.

### 2.6. Statistical Analysis

Statistical analyses were performed using IBM SPSS Statistics for Windows, version 25.0 (IBM Corp., Armonk, NY, USA). The distribution of continuous variables was assessed using the Shapiro–Wilk test.

Normally distributed variables were expressed as mean ± standard deviation, whereas non-normally distributed variables were presented as median (interquartile range [IQR]). Comparisons between OFC-positive and OFC-negative groups were performed using the independent samples t-test or the Mann–Whitney U test, as appropriate.

Categorical variables, including the temporal distribution of patient recruitment (2019–2024), were compared using the chi-square test or Fisher’s exact test when appropriate. Associations between continuous variables were evaluated using Pearson correlation analysis.

The discriminatory performance of biomarkers was evaluated using receiver operating characteristic (ROC) curve analysis. The area under the curve (AUC), sensitivity, specificity, and optimal cutoff values were determined using the Youden index.

To identify independent predictors of OFC positivity, multivariable logistic regression analysis was performed. Variables with *p* < 0.10 in univariable analysis were included in the multivariable model. Results were reported as odds ratios (ORs) with 95% confidence intervals (CIs).

To account for multiple comparisons, Bonferroni correction was applied to the univariable comparisons of laboratory variables and the correlation analyses, each involving seven simultaneous statistical tests. Accordingly, statistical significance was defined as a two-sided *p* value < 0.007 (0.05/7) for these analyses. For all other analyses, a two-sided *p* value < 0.05 was considered statistically significant.

### 2.7. Ethical Approval

This study was approved by the Non-Interventional Clinical Research Ethics Committee of Fırat University (session date: 15 January 2026; decision number: 2026/01-05; document number: 22.01.2026-43706). The study was conducted in accordance with the principles of the Declaration of Helsinki.

## 3. Results

### 3.1. Study Population and Baseline Characteristics

The final study cohort comprised 126 pediatric patients, of whom 66 (52.4%) had a positive OFC result and 60 (47.6%) had a negative result. The cohort included 85 boys (67.5%) and 41 girls (32.5%), with a mean age of 44.5 ± 43.4 months at the time of evaluation. The temporal distribution of patient recruitment was comparable between the OFC-positive and OFC-negative groups (Pearson χ^2^ = 6.94, *p* = 0.226) (Table 2).

Regarding presenting symptoms, urticaria, atopic dermatitis, and other symptoms were observed in 23, 30, and 7 patients in the OFC-negative group, respectively, compared with 36, 20, and 10 patients in the OFC-positive group. There was no statistically significant difference between the groups in terms of symptom distribution (*p* = 0.077). Similarly, sex distribution was comparable between the groups (*p* = 0.842) (Table 3).

### 3.2. Comparison of Clinical and Laboratory Parameters Between OFC Groups

When the OFC-negative group (*n* = 60) was compared with the OFC-positive group (*n* = 66), patients with positive OFC results had significantly larger cow’s milk skin prick test (SPT) wheal diameters and significantly higher casein-specific IgE, SPT-to-histamine ratio, cow’s milk-specific IgE, and total IgE levels (all *p* < 0.01) (Table 4). In contrast, age did not differ significantly between the groups (median [IQR]: 18 [12–36] vs. 36 [18–72] months, *p* = 0.157). The mean SPT wheal diameter was 7.08 ± 2.93 mm in the OFC-positive group compared with 4.85 ± 1.91 mm in the OFC-negative group.

Casein-specific IgE levels were markedly higher in the OFC-positive group (23.99 ± 31.58 kUA/L) compared with the OFC-negative group (1.63 ± 2.73 kUA/L). Similarly, the SPT-to-histamine ratio was significantly higher in OFC-positive patients (1.14 ± 0.54 vs. 0.71 ± 0.32). Cow’s milk-specific IgE and total IgE levels were also significantly elevated in the OFC-positive group (both *p* < 0.01). In contrast, histamine wheal diameter and the specific IgE/total IgE ratio did not differ significantly between the groups (*p* = 0.221 and *p* = 0.266, respectively).

### 3.3. Correlation Analysis

Pearson correlation analysis demonstrated that cow’s milk SPT wheal diameter was positively associated with age, casein-specific IgE level, cow’s milk-specific IgE level, and the SPT-to-histamine ratio. The strongest correlation was observed between SPT wheal diameter and the SPT-to-histamine ratio (r = 0.814, *p* < 0.001), followed by cow’s milk-specific IgE level (r = 0.640, *p* < 0.001), age (r = 0.580, *p* < 0.001), and casein-specific IgE level (r = 0.528, *p* < 0.001) (Table 5).

By contrast, histamine wheal diameter, total IgE level, and the specific IgE/total IgE ratio were not significantly correlated with SPT wheal diameter (*p* > 0.05 for all).

### 3.4. Discriminatory Performance of Casein-Specific IgE

**Figure 1:**
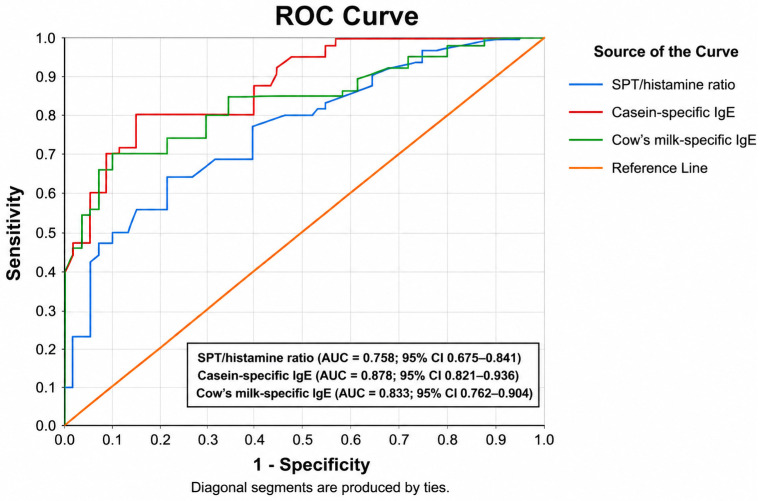
Receiver operating characteristic (ROC) curves of the SPT/histamine ratio, casein-specific IgE, and cow’s milk-specific IgE for predicting positive oral food challenge (OFC) results. The corresponding AUC values were 0.758 (95% CI: 0.675–0.841), 0.878 (95% CI: 0.821–0.936), and 0.833 (95% CI: 0.762–0.904), respectively.

Casein-specific IgE demonstrated strong discriminatory performance for predicting OFC positivity, with an area under the receiver operating characteristic (ROC) curve (AUC) of 0.878 (95% CI: 0.821–0.936; *p* < 0.001; *n* = 126). According to the Youden index, the optimal cutoff value was ≥2.55 kUA/L, yielding a sensitivity of 80.3% and a specificity of 85.0% (Figure 1).

### 3.5. Discriminatory Performance of the SPT-to-Histamine Ratio

The SPT-to-histamine ratio showed moderate discriminatory ability for distinguishing OFC-positive from OFC-negative patients, with an area under the receiver operating characteristic (ROC) curve (AUC) of 0.758 (95% CI: 0.675–0.841; *p* < 0.001; *n* = 126). The optimal cutoff value determined by the Youden index was ≥0.832, corresponding to a sensitivity of 63.6% and a specificity of 78.3% (Figure 1).

### 3.6. Multivariable Predictive Modeling

Multivariable logistic regression analysis showed that the overall model was statistically significant and well calibrated (Hosmer–Lemeshow *p* = 0.600; Nagelkerke R^2^ = 0.494). Among the variables included in the model, only casein-specific IgE remained independently associated with OFC positivity (adjusted OR = 1.317; 95% CI: 1.140–1.522; *p* < 0.001) (Table 6).

By contrast, the cow’s milk-specific IgE level and the SPT-to-histamine ratio lost statistical significance after adjustment (*p* > 0.05 for both).

## 4. Discussion

The most important finding of the present study is that casein-specific IgE was the strongest independent predictor of OFC positivity among children evaluated for suspected cow’s milk allergy. Although the SPT-to-histamine ratio demonstrated significant discriminatory capacity, it did not remain independently associated with challenge positivity after adjustment for other variables. Taken together, these findings suggest that component-resolved diagnostics may provide clinically meaningful added value, particularly in pre-challenge risk stratification and patient selection. In line with current guideline recommendations, clinical history, skin prick testing, and serum-specific IgE remain the cornerstone of the diagnostic approach, whereas OFC continues to represent the reference standard for confirming clinical reactivity, particularly in diagnostically uncertain cases [5,6,7,10].

Casein-specific IgE showed strong discriminatory performance on ROC analysis and retained its independent predictive value in the multivariable model, underscoring the clinical relevance of component-resolved diagnostics in cow’s milk allergy. Previous systematic reviews and meta-analyses have demonstrated that extract-based SPT and cow’s milk-specific IgE testing are generally associated with higher sensitivity, whereas component-specific IgE measurements provide superior specificity; notably, casein-specific IgE has been reported to achieve a specificity of approximately 93% [5]. In line with this, Ayats-Vidal et al. reported that casein-specific IgE yielded the best performance for predicting a positive OFC result, with an AUC of 0.976 [6]. Likewise, Tosca et al. identified casein-specific IgE as a significant predictive marker and proposed a cutoff value of 4.87 kUA/L [7]. The cutoff identified in our cohort (2.55 kUA/L) appears generally compatible with these observations. Nevertheless, such thresholds should be interpreted cautiously, as they may vary according to patient characteristics, age distribution, and assay methodology. Accordingly, validation in independent external cohorts is required before this cutoff can be incorporated into routine clinical decision-making.

By contrast, although the SPT-to-histamine ratio demonstrated significant discriminatory ability for OFC positivity, it did not retain independent predictive value in the multivariable model. This observation suggests that, as an SPT-derived index normalized to the histamine response, it may largely reflect biological information already captured by conventional SPT measurements. This interpretation is supported by the strong positive correlation between SPT wheal diameter and the SPT-to-histamine ratio (r = 0.814). Furthermore, the greater specificity and stronger biological signal of casein-specific IgE may explain why the ratio lost significance after adjustment. Taken together, these findings indicate that histamine-normalized indices are not without clinical relevance, but are more appropriately regarded as supportive adjunctive markers rather than stand-alone determinants in decision-making [8,11].

Several limitations of this study should be acknowledged. First, the retrospective single-center design may have introduced selection bias and limits the generalizability of the findings. Although the temporal distribution of patient recruitment was comparable between the OFC-positive and OFC-negative groups throughout the study period, recruitment overlapped with the COVID-19 pandemic. Changes in healthcare utilization during the pandemic may have influenced referral patterns, and therefore some degree of pandemic-related selection bias cannot be completely excluded. Second, the proposed cutoff values may be center- and assay-specific and therefore require external validation in independent cohorts before they can be broadly implemented in clinical practice. In addition, component-resolved diagnostics were limited to casein-specific IgE, while other major cow’s milk allergen components, including α-lactalbumin (Bos d 4) and β-lactoglobulin (Bos d 5), were not evaluated. The inclusion of these component-specific IgE measurements might have provided additional information regarding sensitization profiles and their potential contribution to predicting OFC outcomes. Furthermore, because OFC was performed according to clinical indication rather than uniformly across all sensitized patients, the pre-test probability of the study population may have influenced the observed diagnostic performance. Although delayed eczema exacerbation was predefined as a secondary outcome, its retrospective assessment from medical records was insufficiently standardized to allow a robust analysis. Therefore, the interpretation of the present findings should primarily rely on immediate objective OFC positivity. Finally, histamine-normalized indices remain insufficiently standardized in the current literature, underscoring the need for prospective multicenter studies to validate their reproducibility and clinical applicability.

Despite these limitations, the study has several notable strengths. It simultaneously evaluated conventional SPT parameters, histamine response, histamine-normalized indices, casein-specific IgE, cow’s milk-specific IgE, and total IgE within the same well-characterized pediatric cohort, while using OFC as the reference standard for confirming clinical reactivity. This comprehensive approach enabled direct comparison of multiple diagnostic markers under identical clinical conditions and provides clinically relevant evidence supporting the value of casein-specific IgE and histamine-normalized SPT indices in the diagnostic assessment of suspected cow’s milk allergy.

## 5. Conclusions

In conclusion, the present findings support casein-specific IgE as a robust and clinically relevant biomarker for predicting OFC positivity in children with suspected cow’s milk allergy. Although the SPT-to-histamine ratio appears to provide complementary diagnostic information, its independent predictive contribution seems to be weaker than that of casein-specific IgE. From a clinical perspective, casein-specific IgE may aid pre-challenge risk stratification, facilitate more rational patient selection, and potentially reduce unnecessary elimination diets and challenge procedures. This may be particularly relevant in early childhood, where prolonged dietary restrictions can adversely affect nutritional intake, growth, and quality of life. Therefore, improved prediction of OFC outcomes may contribute not only to diagnostic decision-making but also to more appropriate nutritional management in pediatric patients. However, broader multicenter studies with external validation are needed before this approach can be incorporated into routine diagnostic algorithms. Importantly, no biomarker should be considered a substitute for OFC, and biomarker-based interpretation remains meaningful only within the context of careful clinical history taking and expert clinical judgment.

## Figures and Tables

**Table 1:** Age-adapted stepwise dose escalation protocol used for oral food challenge (OFC) with cow’s milk.

Step	Administered Dose (mL)	Cumulative Dose (mL)
(**A**) <1 year (infant formula; target cumulative dose: 100–150 mL)		
1	0.5	0.5
2	1.5	2
3	5	7
4	15	22
5	30	52
6	48–98	100–150
(**B**) ≥1 year (pasteurized cow’s milk; target cumulative dose: 200–220 mL)		
1	0.5	0.5
2	1.5	2
3	5	7
4	15	22
5	50	72
6	128–148	200–220

Doses were administered at 20–30 min intervals. The protocol was adapted from the semi-logarithmic dose escalation model recommended in the EAACI/PRACTALL guidelines, and tailored to age-specific target cumulative volumes. In high-risk patients, lower starting doses, slower dose escalation, and additional intermediate steps were applied when necessary.

**Table 2:** Temporal distribution of patient recruitment according to oral food challenge (OFC) outcome.

Year of Recruitment	OFC-Negative (*n* = 60) *n* (%)	OFC-Positive (*n* = 66) *n* (%)	*p*-Value
2019	14 (23.3)	6 (9.1)	
2020	11 (18.3)	13 (19.7)	
2021	13 (21.7)	11 (16.7)	
2022	5 (8.3)	9 (13.6)	
2023	12 (20.0)	17 (25.8)	
2024	5 (8.3)	10 (15.2)	
Overall comparison			0.226

Data are presented as *n* (%). Comparison was performed using Pearson’s chi-square test.

**Table 3:** Baseline demographic and clinical characteristics according to OFC outcome.

Variable	OFC-Negative (*n* = 60)	OFC-Positive (*n* = 66)	*p*-Value
**Demographics**			
Age (months), median (IQR)	18 (12–36)	36 (18–72)	0.157
Sex, *n* (%)			0.842
Male	41 (68.3)	44 (66.7)	
Female	19 (31.7)	22 (33.3)	
**Clinical presentation**			
Presenting symptom, *n* (%)			0.077
Urticaria	23 (38.3)	36 (54.5)	
Atopic dermatitis	30 (50.0)	20 (30.3)	
Other	7 (11.7)	10 (15.2)	

Data are presented as mean ± SD or *n* (%). OFC, oral food challenge; SD, standard deviation.

**Table 4:** Comparison of skin prick test and laboratory parameters according to oral food challenge (OFC) outcome.

Variable	OFC-Negative (*n* = 60) Mean ± SD	OFC-Positive (*n* = 66) Mean ± SD	*p*-Value
Skin prick test wheal diameter (mm)	4.85 ± 1.91	7.08 ± 2.93	<0.001
Histamine wheal diameter (mm)	7.09 ± 1.36	6.72 ± 2.00	0.221
Casein-specific IgE (kUA/L)	1.63 ± 2.73	23.99 ± 31.58	<0.001
SPT/histamine ratio	0.71 ± 0.32	1.14 ± 0.54	<0.001
Cow’s milk-specific IgE (kUA/L)	4.18 ± 4.91	34.07 ± 35.85	<0.001
Total IgE (IU/mL)	128.42 ± 281.99	911.17 ± 2191.11	0.005
Specific IgE/Total IgE ratio	0.27 ± 0.72	0.16 ± 0.22	0.266

SPT, skin prick test; SD, standard deviation; IgE, immunoglobulin E; OFC, oral food challenge.

**Table 5:** Pearson correlations between cow’s milk skin prick test wheal diameter and other variables.

Variable	Pearson r	*p*-Value
Age (months)	0.580	<0.001
Histamine wheal diameter	−0.004	0.966
Casein-specific IgE	0.528	<0.001
SPT/histamine ratio	0.814	<0.001
Cow’s milk-specific IgE	0.640	<0.001
Total IgE	0.096	0.284
Specific IgE/Total IgE ratio	−0.064	0.475

**Table 6:** Multivariable logistic regression analysis for predictors of OFC positivity.

Variable	Adjusted OR	95% CI	*p*-Value
Casein-specific IgE	1.317	1.140–1.522	<0.001

OR, odds ratio; CI, confidence interval; OFC, oral food challenge. Model χ^2^ = 58.286, *p* < 0.001; Nagelkerke R^2^ = 0.494; Hosmer–Lemeshow *p* = 0.600.

## Data Availability

The data presented in this study are available on reasonable request from the corresponding author. The data are not publicly available due to patient privacy concerns and ethical restrictions.
